# Laparoscopic electrochemotherapy for the treatment of hepatocellular carcinoma: Technological advancement

**DOI:** 10.3389/fonc.2022.996269

**Published:** 2022-11-10

**Authors:** Blaz Trotovsek, Benjamin Hadzialjevic, Maja Cemazar, Gregor Sersa, Mihajlo Djokic

**Affiliations:** ^1^ Department of Abdominal Surgery, University Medical Center Ljubljana, Ljubljana, Slovenia; ^2^ Department of Surgery, Faculty of Medicine, University of Ljubljana, Ljubljana, Slovenia; ^3^ Department of Experimental Oncology, Institute of Oncology, Ljubljana, Slovenia; ^4^ Faculty of Health Sciences, University of Primorska, Izola, Slovenia; ^5^ Faculty of Health Sciences, University of Ljubljana, Ljubljana, Slovenia

**Keywords:** electrochemotherapy, hepatocellular carcinoma, minimally invasive, laparoscopy, treatment, immunotherapy

## Abstract

Electrochemotherapy is an effective treatment modality for hepatocellular carcinoma (HCC). Electrochemotherapy for HCC was initially used in the setting of open surgery. Recently, with the development of newer electrodes, percutaneous approaches have also been performed. However, laparoscopic application of electrochemotherapy for HCC has not yet been described. Two patients with unresectable HCC were enrolled in the study. The first patient was not suitable for the percutaneous approach because the tumor was located close to the gallbladder. He also had symptomatic gallstones. The second patient had HCC in close proximity to the stomach and was therefore not suitable for percutaneous access or any other ablative technique. Thus, the laparoscopic approach was chosen, using newly developed Stinger electrodes for the application of electric pulses. After intravenous administration of bleomycin, several sets of electric pulses were delivered to the whole tumor mass in both patients. Ultrasonographically, the coverage of the whole tumor was verified, as described previously. Cholecystectomy was also performed in the first patient. Follow-up abdominal computed tomography showed a complete response of the treated lesions in both patients. Minimally invasive laparoscopic electrochemotherapy is safe, feasible and effective method for the treatment of HCC. It could be used in patients in whom the percutaneous approach is unsafe (proximity to other organs) and in patients with concomitant symptomatic gallstones in whom cholecystectomy is already indicated. This technological approach thus allows broader and minimally invasive clinical applicability of electrochemotherapy.

## Introduction

Liver cancer is the sixth most common cancer, with more than 800,000 new cases in 2018, and the fourth leading cause of cancer-related death worldwide (1). Hepatocellular carcinoma (HCC) accounts for approximately 75% of liver cancers (2). HCC is projected to be the third leading cause of cancer-related death by 2030 (3). The vast majority of cases of HCC occur in association with chronic liver disease or liver cirrhosis. The strongest risk factors for liver cirrhosis are extensive alcohol abuse, nonalcoholic fatty liver disease (NAFLD) due to obesity, and chronic hepatitis B/C virus infection (4).

Complete resection of HCC by partial hepatectomy or by total hepatectomy and liver transplantation is the curative treatment of choice with the highest overall survival (5). However, because the vast majority of HCC cases occur in patients with cirrhosis and other comorbidities, treatment (especially aggressive surgical resection) could be limited based on the patient’s overall health and liver function. Therefore, other local ablative methods may be used (6). For example, in early-stage HCC, radiofrequency ablation (RFA) and microwave ablation (MWA) are accepted as viable treatments for smaller tumors or as alternatives to surgery for larger tumors. Locoregional modalities such as transarterial chemoembolization (TACE) or transarterial radioembolization (TARE) are used in intermediate-stage HCC (liver-confined, multinodular disease) in patients with Child–Pugh class A or B cirrhosis and in the absence of portal vein invasion. Patients with Child–Pugh class C cirrhosis and early-stage HCC should be first considered for liver transplantation (1, 7).

Electrochemotherapy is an emerging method for the treatment of cutaneous and deep-seated tumors (8–10). Electrochemotherapy has been shown to be safe and effective in the treatment of HCC in the setting of open surgery (11, 12). Recently, with the development of newer electrodes, percutaneous approaches to electrochemotherapy have also been described (13). Furthermore, percutaneous electrochemotherapy has been shown to be feasible, safe, and effective in the treatment of HCC (14). However, in cases where tumors are located in close proximity to other organs, the percutaneous approach is considered unsafe (15). Therefore, laparoscopic electrochemotherapy may be beneficial as a minimally invasive approach in these patients. To the best of our knowledge, no cases of laparoscopic electrochemotherapy for HCC have been described to date.

In this case-controlled study, the authors aimed to test the feasibility, safety, and efficacy of laparoscopic electrochemotherapy in the treatment of HCC.

## Patients, methods and results

**Figure 1:**
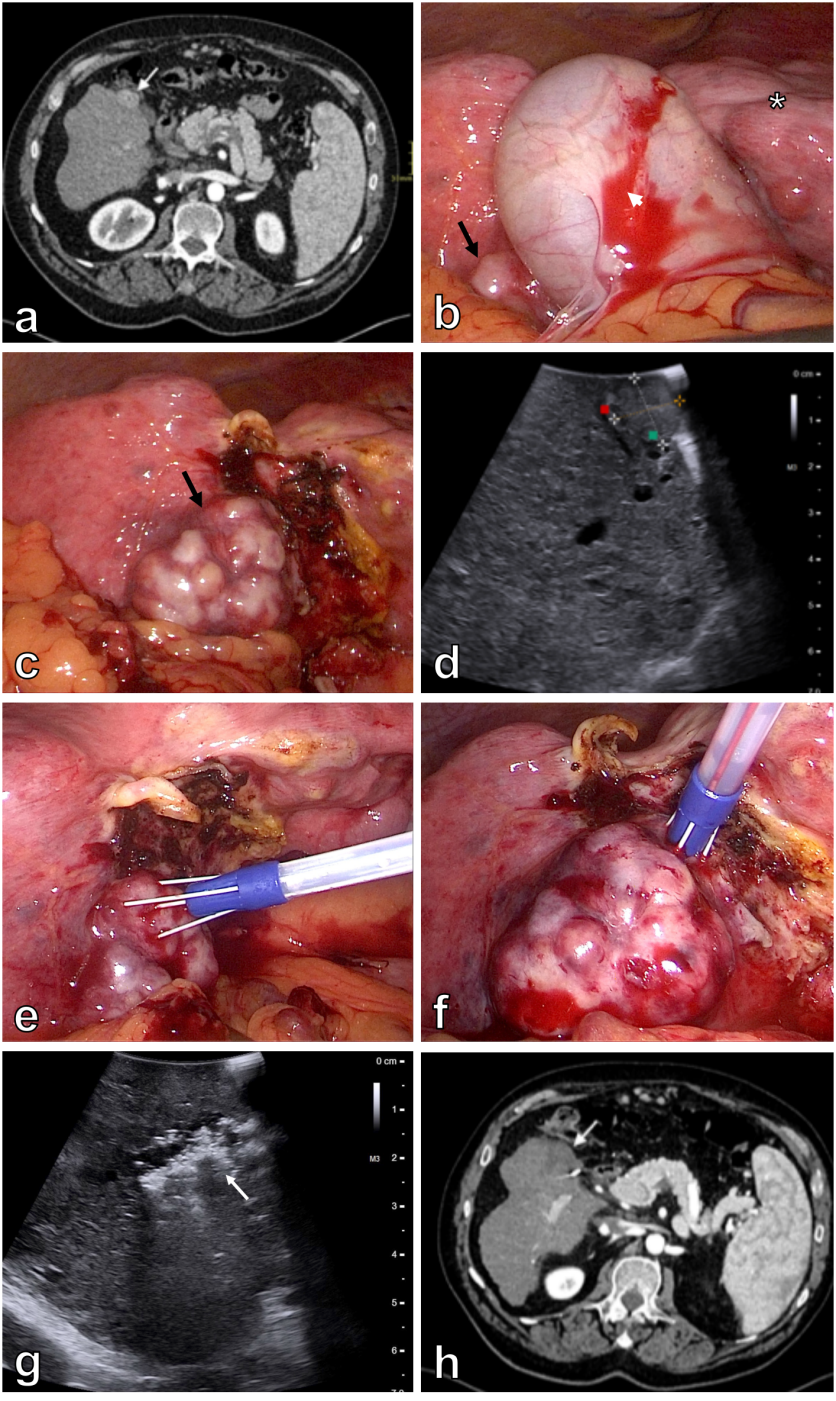
Patient 1. Initial contrast enhanced abdominal CT demonstrates a 12 x 18 mm hypervascular lesion in segment V of the cirrhotic liver (white arrow) **(A)**. Laparoscopy shows macronodular liver cirrhosis (asterisk) with an exulcerated tumor in segment V (black arrow). The majority of the tumor is covered with gallbladder (white arrowhead) **(B)**. Optimal exposure of the tumor (black arrow) is achieved after cholecystectomy **(C)**. Intraoperative US better demonstrates a 22 mm lesion in segment V of the liver **(D)**. Electric pulses are applied through 10° divergent Stinger electrode inserted in the center of the tumor **(E)** and around the tumor **(F)**. Control intraoperative US immediately after the treatment demonstrates area of avital lesion (white arrow) **(G)**. Contrast enhanced abdominal CT four months after the procedure demonstrates a 35 mm area of ablation necrosis in segment V of the liver **(H)**.

Two patients were enrolled in the study. Both patients were not suitable for other curative treatments according to Barcelona Clinic Liver Cancer (BCLC) classification. Patient 1 was a 64-year-old man with Child–Pugh class B liver cirrhosis – ethylic etiology, aortic stenosis, and symptomatic gallstone disease. He had a history of variceal bleeding and multiple recurrent episodes of hepatic encephalopathy. Patient was presented at a hepatobiliary multidisciplinary team meeting with a 12 x 18 mm HCC in segment V, which was located in close proximity to the gallbladder according to abdominal computed tomography (CT) (**Figure 1A**). In addition, no signs of extrahepatic disease were found. Initially, TACE was indicated. However, the first attempt of TACE was unsuccessful because of dissection of the right hepatic artery. At abdominal follow-up CT one month after the first procedure, the right hepatic artery was recanalized. Therefore, a second attempt at TACE was initiated. Selective catheterization and cone beam computed tomography (CBCT) during the procedure showed that the tumor was fed by the cystic artery. TACE was therefore not possible, as it would lead to necrosis of the gallbladder. Thus, the patient’s records were again reviewed at a hepatobiliary multidisciplinary team meeting, in which laparoscopic electrochemotherapy with laparoscopic cholecystectomy was indicated.

**Figure 2:**
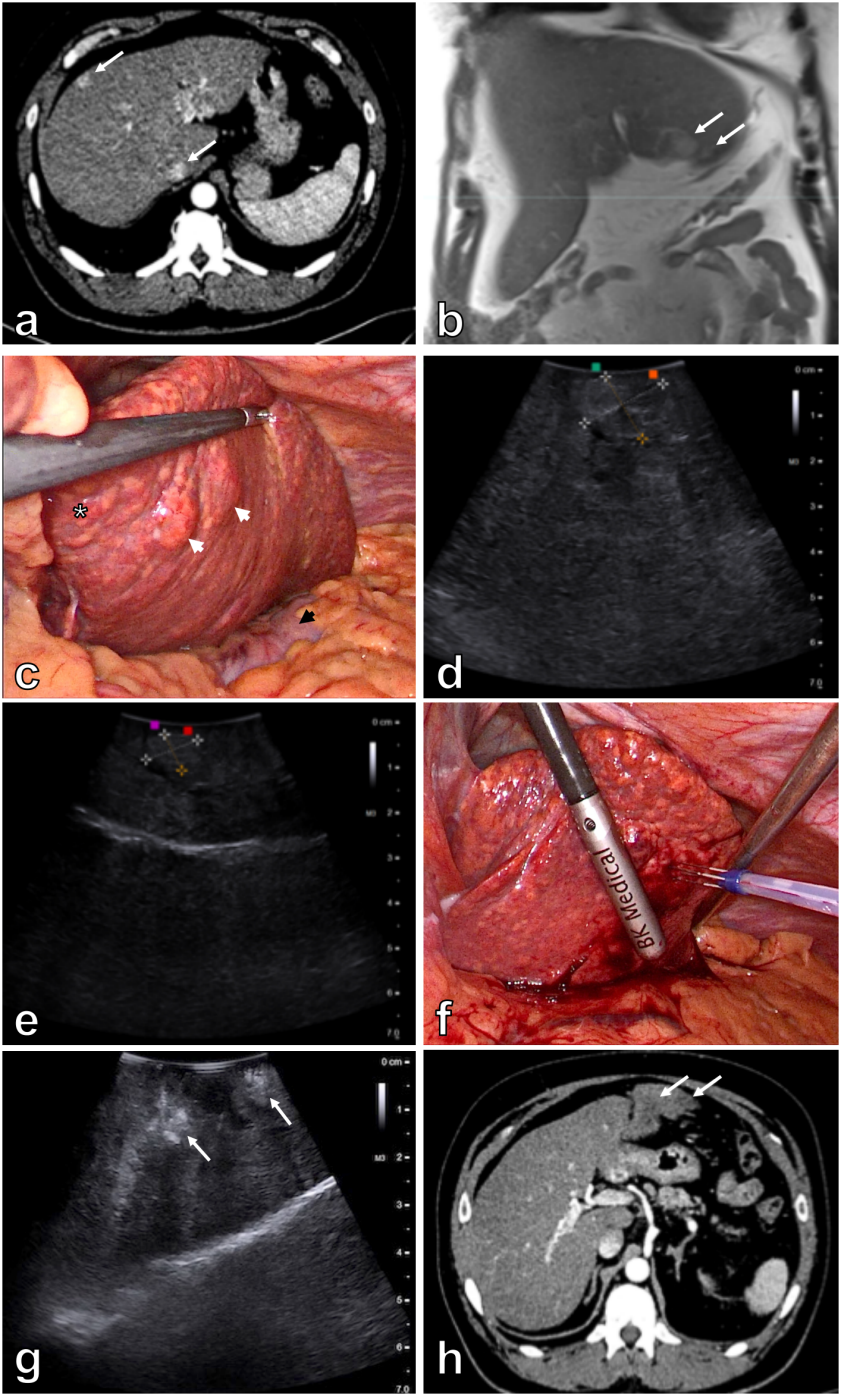
Patient 2. Initial contrast enhanced abdominal CT demonstrates multiple hypervascular lesions (white arrows) in the cirrhotic liver **(A)**. Contrast enhanced MRI of the liver after 24 cycles of immunotherapy demonstrates 18 mm and 8 mm lesions (white arrows) in segment III of the liver **(B)**. Laparoscopy shows macronodular liver cirrhosis (asterisk). Both tumors (white arrowheads) are seen after the retraction of the left liver lobe. Notice the stomach just underneath the left liver lobe (black arrowhead) **(C)**. Intraoperative US better demonstrates a larger 20 x 15 mm tumor **(D)** and a smaller 8 x 12 mm tumor in the segment III **(E)**. Electric pulses to the tumor are applied through convergent Stinger electrode. Placement of the electrode is aided by intraoperative US **(F)**. Control intraoperative US immediately after the treatment demonstrates areas of avital lesions (white arrows) **(G)**. Contrast enhanced abdominal CT three months after the procedure demonstrates 22 mm and 10 mm avascular lesions (white arrows) in segment III of the liver **(H)**.

Patient 2 was a 43-year-old man with Child–Pugh class A liver cirrhosis – NAFLD etiology, obesity, arterial hypertension, and type 2 diabetes. The patient was initially diagnosed with multifocal HCC in segments II, III, IVa, and VIII (**Figure 2A**). Thus, immunotherapy with atezolizumab and bevacizumab was initiated. He received 24 cycles of immunotherapy. A control magnetic resonance imaging (MRI) scan of the upper abdomen showed a good response to the treatment, as only two lesions were seen in segment III, which were located in close proximity to the stomach (**Figure 2B**). In addition, no signs of extrahepatic disease were found. His records were presented at a hepatobiliary multidisciplinary team meeting. According to BCLC algorithm the patient was not suitable for surgical resection because of portal hypertension. Thus, laparoscopic electrochemotherapy for the two remaining lesions was recommended.

The clinical study (NCT02291133) was approved by the National Ethics Committee (21k/02/14). Both patients signed an informed consent form. Laparoscopic electrochemotherapy was performed according to the updated standard operating procedures for electrochemotherapy and according to the previous study protocol for electrochemotherapy in HCC during open surgery (11, 16).

The procedures were performed at the Clinical Department of Abdominal Surgery, University Medical Center Ljubljana. The procedures were performed under general anesthesia. Both patients were positioned in a reverse Trendelenburg split-leg (French) position, with the surgeon standing between the patient’s legs and the assistant on the patient’s left side. A pneumoperitoneum of 11 mmHg was established using a Veress needle technique in the umbilical region and trocars were placed as follows: 11 mm trocar supraumbilically, 11 mm trocar in the epigastrium, and two 5 mm trocars in the right upper quadrant.

Laparoscopy in the first patient revealed macronodular liver cirrhosis. The tumor was located in segment V of the liver, in close proximity to the gallbladder (**Figure 1B**). To better visualize the tumor and safely perform electrochemotherapy, removal of the gallbladder was initiated. Thus, laparoscopic cholecystectomy was safely performed, and the whole tumor was exposed (**Figure 1C**). Intraoperative ultrasound (BK Medical, Burlington, USA) confirmed the tumor in segment V, measuring 22 mm in the longer axis (**Figure 1D**). Ten-degree divergent Stinger electrode (IGEA SpA, Carpi, Italy) was positioned into and around the tumor, aided by intraoperative ultrasound (**Figures 1E–F**). Electric pulses were delivered by an electric pulse generator (IGEA SpA, Carpi, Italy) during an interval of 8–28 minutes after bolus intravenous injection of bleomycin 15,000 IU/m^2^ (dose per patient 29,900 IU; Bleomycin medac, Medac, Germany). A total of 20 electric pulses were delivered to cover the whole tumor mass, including the safety margins (**Table 1**). Patient safety was achieved by synchronizing the delivery of the electric pulses with the absolute refractory period of the heart (17). Control intraoperative ultrasound immediately after the extraction of the electrode revealed area of avital lesion (**Figure 1G**).

**Table 1:** Patients, procedures, electrodes and response to the treatment.

	Patient 1	Patient 2
No of treated lesions	1	2
No of applications per lesion/per patient	20/20	16 + 39/55
Amplitude	1000 V	1000 V
Time length of pulses	100 μs	100 μs
Frequency of pulses	5 kHz	5 kHz
Pulses per sequence	160	160
Stinger electrode	10° divergent electrode	Convergent electrode
Needle diameter/length/active part	0.45 mm/40 mm/15 mm	0.45 mm/40 mm/20 mm
Follow-up	Complete response	Complete response

Laparoscopy in patient 2 also revealed macronodular liver cirrhosis. Two tumors were noted in segment III of the liver in close proximity to the stomach (**Figure 2C**). Tumors measured 20 x 15 mm and 8 x 12 mm in the longer axis according to intraoperative ultrasound (**Figures 2D, E**). Convergent Stinger electrode was positioned into and around both tumors, aided by intraoperative ultrasound (**Figure 2F**). Electric pulses were delivered by an electric pulse generator during an interval of 8–28 minutes after bolus intravenous injection of bleomycin 15,000 IU/m^2^ (dose per patent 30,000 IU). To the first tumor (8 x 12 mm) a total of 16 electric pulse applications and to the second one (20 x 15 mm) 39 applications of electric pulses to cover both tumors including the safety margins (**Table 1**). Patient safety was achieved by synchronizing the delivery of the electric pulses with the absolute refractory period of the heart (17). Control intraoperative ultrasound revealed areas of avital lesions (**Figure 2G**).

The postoperative period was uneventful in both patients. Patient 1 was discharged on the 5^th^ postoperative day, and patient 2 was discharged on the 2^nd^ postoperative day. Follow-up abdominal CT in patient 1 performed after one and four months showed a 35 mm area of ablation necrosis in segment V of the liver within the treated area (**Figure 1H**). A control abdominal CT in patient 2 three months after the procedure also showed 22 mm and 10 mm avascular lesions in segment III of the liver within the treated area (**Figure 2H**). Thus, a complete response of the targeted lesions was observed in both patients. Both patients continue to adhere to surveillance.

## Discussion

We have described the first two cases of laparoscopic electrochemotherapy for HCC. Laparoscopic electrochemotherapy was found to be feasible, safe, and effective for the treatment of unresectable HCC.

The treatment of HCC has improved in recent years. According to the 2018 modified BCLC classification of the European Association for the Study of Liver (EASL), patients with HCC are classified into different stages (18). In general, patients with early-stage HCC are candidates for resection, liver transplantation, or local ablation, whereas patients with intermediate to advanced-stage HCC are candidates for TACE or systemic therapy (1, 18). In our study, patient 1 could be assigned to the group of patients with early-stage HCC because he had Child–Pugh class B cirrhosis and a solitary lesion smaller than 3 cm. However, he was an active alcohol abuser and was therefore not a candidate for liver transplantation. In addition, percutaneous methods were contraindicated because the tumor was in close proximity to the gallbladder, whereas TACE was not feasible as the tumor was supplied by the cystic artery. Patient 2 was initially diagnosed with multifocal HCC and was therefore treated with immunotherapy. After 24 cycles of immunotherapy, only two vital HCC lesions remained in the liver. However, according to the BCLC algorithm he was not an optimal candidate for surgical resection because of portal hypertension. Similar to the first patient, he was neither a candidate for percutaneous methods because both HCCs were in close proximity to the stomach.

Electrochemotherapy is an effective, locoregional ablative treatment modality. Basically, electric pulses delivered to the target lesion increase the permeability of the tumor’s cell membrane, thus facilitating the intracellular delivery of the chemotherapeutic agent (mainly bleomycin and cisplatin). Thus, the cells are dying by apoptosis or other programmed types of cell death. In addition, the doses required for the optimal cytotoxic effect of chemotherapeutic agents are lower, which eventually results in fewer systemic side effects (10, 19–21). Moreover, electrochemotherapy has been shown to be safe in the treatment of tumors in close proximity to the major vessels, as no significant damage to the vascular structures of the liver has been observed in previous studies in pigs (22, 23).

The clinical application of electrochemotherapy was initially investigated in patients with superficial tumors and is now used in more than 170 centers worldwide for the treatment of cutaneous tumors as an alternative to surgical resection (24, 25). The transition to the treatment of deep-seated tumors and the technological approach were first described in a pilot and a subsequent phase 2 study on colorectal liver metastases. In both studies, electrochemotherapy proved to be a safe and effective treatment modality (26, 27). Electrochemotherapy was also shown to be safe and effective in the treatment of patients with HCC where other treatment modalities have been exhausted. Complete response rates were achieved in 80% of patients and in 88% of treated lesions, while no adverse effects or major postoperative complications were observed. A transient increase in liver enzymes and bilirubin was commonly observed, while transient liver dysfunction with ascites formation occurred in only 4 of 24 patients, which resolved spontaneously in two patients and after diuretic therapy in the other two patients (11, 12).

Technological advances and the development of newer electrodes and pulse generators have eventually enabled the minimally invasive, percutaneous application of electrochemotherapy. To the best of our knowledge, the first percutaneous electrochemotherapy for the treatment of HCC was performed at the University Medical Center Ljubljana and proved to be feasible, safe, and effective (14). Nevertheless, percutaneous ablative methods should generally be avoided in cases where liver lesions are located superficially in close proximity to the gastrointestinal tract, gallbladder or diaphragm (15). Both patients in our study were not suitable candidates for percutaneous access because the lesions were in close proximity to the gallbladder and stomach. However, laparoscopic or endoscopic approaches have recently become feasible with the newly developed Stinger electrodes. Thus, a novel approach to electrochemotherapy using newly developed Stinger electrode for the treatment of colorectal cancer was recently described (28).

Electrochemotherapy elicits a local immune response and induces immunogenic cell death. Thus, the *in situ* vaccination of electrochemotherapy could be boosted by adjuvant immunogenic therapy (29). A recent study suggested that electrochemotherapy improves superficial tumor control in melanoma patients treated with immunotherapy, leading to longer progression-free survival and overall survival (30). In addition, HCC is a potentially immunogenic tumor. Therefore, electrochemotherapy in combination with immunotherapy may be beneficial in achieving a better and longer-lasting antitumor response in patients with HCC (31). To our knowledge, we report the first case of a patient with HCC who was initially treated with immunotherapy followed by electrochemotherapy for the two remaining lesions. A complete response was observed three months after the electrochemotherapy. However, further follow-up is needed to demonstrate the long-term effect of the combined treatments.

Gallstones are a major health problem in developed countries, affecting up to 20% of the adult population. The majority of patients with gallbladder stones remain asymptomatic, but up to 20% of these patients eventually develop symptoms (32). According to EASL guidelines, symptomatic patients should undergo cholecystectomy (preferably laparoscopic) due to recurrent colic and the higher likelihood of complications associated with gallstones (33). Patient 1 in our study also had symptomatic gallbladder stones and was already a candidate for cholecystectomy. Here, we therefore demonstrated a dual benefit of the laparoscopic approach to electrochemotherapy in the treatment of concurrent HCC and gallbladder stones.

## Conclusion

Minimally invasive laparoscopic electrochemotherapy is safe, feasible and effective method for the treatment of HCC. It could be used in patients in whom the percutaneous approach is unsafe (proximity to other organs) and in patients with concomitant symptomatic gallstones in whom cholecystectomy is already indicated. This technological approach thus allows broader and minimally invasive clinical applicability of electrochemotherapy. In addition, we describe the first case of electrochemotherapy in combination with immunotherapy in a patient with HCC.

## Data availability statement

The raw data supporting the conclusions of this article will be made available by the authors, without undue reservation.

## Ethics statement

The studies involving human participants were reviewed and approved by National Ethics Committee of the Republic of Slovenia The clinical study (NCT02291133) was approved (21k/02/14). The patients/participants provided their written informed consent to participate in this study.

## Author contributions

Conceptualization: BT, MC, GS, and MD; writing – original draft: BH; writing – review and editing: BT, MC, GS, and MD. All authors contributed to the article and approved the submitted version.

## Funding

The authors acknowledge the financial support from the state budget by the Slovenian Research Agency, the program no. P3-0003.

## Conflict of interest

The authors declare that the research was conducted in the absence of any commercial or financial relationships that could be construed as a potential conflict of interest.

## Publisher’s note

All claims expressed in this article are solely those of the authors and do not necessarily represent those of their affiliated organizations, or those of the publisher, the editors and the reviewers. Any product that may be evaluated in this article, or claim that may be made by its manufacturer, is not guaranteed or endorsed by the publisher.
